# Thyroid hormone increases fatty acid use in fetal ovine cardiac myocytes

**DOI:** 10.14814/phy2.15865

**Published:** 2023-11-27

**Authors:** Natasha Chattergoon, Samantha Louey, Sonnet S. Jonker, Kent L. Thornburg

**Affiliations:** ^1^ Center for Developmental Health, Knight Cardiovascular Institute Oregon Health & Science University Portland Oregon USA

**Keywords:** fetus, heart, heart metabolism, lipid metabolism

## Abstract

Cardiac metabolic substrate preference shifts at parturition from carbohydrates to fatty acids. We hypothesized that thyroid hormone (T_3_) and palmitic acid (PA) stimulate fetal cardiomyocyte oxidative metabolism capacity. T_3_ was infused into fetal sheep to a target of 1.5 nM. Dispersed cardiomyocytes were assessed for lipid uptake and droplet formation with BODIPY‐labeled fatty acids. Myocardial expression levels were assessed PCR. Cardiomyocytes from naïve fetuses were exposed to T_3_ and PA, and oxygen consumption was measured with the Seahorse Bioanalyzer. Cardiomyocytes (130‐day gestational age) exposed to elevated T_3_ in utero accumulated 42% more long‐chain fatty acid droplets than did cells from vehicle‐infused fetuses. In utero T_3_ increased myocardial mRNA levels of CD36, CPT1A, CPT1B, LCAD, VLCAD, HADH, IDH, PDK4, and caspase 9. In vitro exposure to T_3_ increased maximal oxygen consumption rate in cultured cardiomyocytes in the absence of fatty acids, and when PA was provided as an acute (30 min) supply of cellular energy. Longer‐term exposure (24 and 48 h) to PA abrogated increased oxygen consumption rates stimulated by elevated levels of T_3_ in cultured cardiomyocytes. T_3_ contributes to metabolic maturation of fetal cardiomyocytes. Prolonged exposure of fetal cardiomyocytes to PA, however, may impair oxidative capacity.

## 1 INTRODUCTION

Fetal cardiomyocytes undergo a dramatic intracellular maturation process in preparation for metabolizing fatty acids from milk after birth (Bartelds et al., 2000; Fisher et al., 1980; Lopaschuk & Jaswal, 2010). The myocardium of fetal mammals uses carbohydrates like lactate, glucose and pyruvate as primary fuels, generating ATP through aerobic glycolysis. By the time of birth, the myocardium is ready to make a dramatic switch to oxidative phosphorylation, as mitochondria in the mature heart begin to generate ATP for contraction primarily through beta oxidation of free fatty acids (Lopaschuk et al., 2021; Lopaschuk & Jaswal, 2010; Persad & Lopaschuk, 2022; Stanley et al., 2005).

The complex features that regulate this fuel preference switch are not fully understood. However, we and others have investigated maturational switches in the sheep model. In humans and sheep, circulating tri‐iodo‐l‐thyronine (T_3_) increases in fetuses in the immediate prepartum period as thyroxin (T_4_) is de‐iodinated by increasing levels of deiodinases that are upregulated under the stimulation of increasing levels of cortisol (Fowden et al., 2022; Jonker & Louey, 2016). This increase in plasma T_3_ suppresses proliferation of cardiomyocytes, stimulates their binucleation, promotes their enlargement, increases expression of Ca^++^ cycling genes, and increases contractile force (Chattergoon, 2019; Graham & Huang, 2021; Jonker & Louey, 2016).

T_3_ has a central role in maturing oxidative phosphorylation in fetal brain, skeletal muscle, and adipose tissue (Fowden et al., 2022; Fowden & Forhead, 2022). In the heart, T_3_ promotes the maturation of the constituent enzymes that regulate beta oxidation and plays a role in tissue glycogen content (Forhead et al., 2009; Fowden et al., 2022; Graham & Huang, 2021; Sayre & Lechleiter, 2012). There is also a suggestion that the presence of fatty acids themselves helps stimulate this maturational process (Yang et al., 2019).

The degree to which the metabolism of free fatty acids such as palmitic acid (PA), the most common saturated fatty acid in the body, is affected by the presence of T_3_ in fetal cardiomyocytes is not known. Further, it is unknown how fatty acids themselves regulate metabolism in immature cardiomyocytes in the presence of T_3_. In this study, we hypothesized that T_3_ elevation in utero augments the free fatty acid uptake and use system in ex vivo fetal cardiomyocytes, and that T_3_ stimulation increases maximal oxidative phosphorylation in cultured cardiomyocytes. We also tested the hypothesis that PA exposure in culture further increases maximal oxygen consumption in immature cardiomyocytes exposed to T_3_.

## 2 METHODS

### 2.1 In utero treatment with T_3_ for live cell imaging

The Institutional Animal Care and Use Committee at Oregon Health & Science University approved all the studies.

Sheep (Ovis aries) assigned to the in vivo study groups had surgery at 119 ± 1 days of gestation (dGA; term = 147 dGA) similarly to surgeries previously described (Davis et al., 2018). Apart from a pre‐surgical fast, food and water were supplied ad libitum. Different from previously, atropine was not given. Pain was managed with subcutaneous buprenorphine (0.3 mg) together with slow‐release buprenorphine (0.05 mg kg^−1^) following extubation. Sixteen ewes bearing singleton fetuses were assigned to surgery, one was discovered to have twin fetuses in surgery, but only one fetus was instrumented for study. In one ewe, the catheters were compromised following surgery and the animal was withdrawn from the study. In a second ewe, the fetus died abruptly mid experiment with indications consistent with cord occlusion. Consequently, each in utero group is comprised of seven fetuses. Distribution of sexes between groups was random and was not different (vehicle‐infusion six females, one male; T_3_‐infusion two females, five males; *p* = 0.1026).

In utero monitoring was as previously described (Davis et al., 2018). Experiments were started at 125 ± 0 dGA after 7 ± 1 day of surgical recovery. Experimental fetuses received a continuous intravenous infusion of T_3_ (54 μg d^−1^; Sigma‐Aldrich, Cat. No. T6397) as previously described to increase within 1 day total plasma T_3_ to a target of 1.5 nM, approximately equal to the level of total T_3_ in the ovine fetus 2 days before birth (Chattergoon, Giraud, et al., 2012; Jonker & Louey, 2016). Control fetuses received the vehicle solution (1.4 mM NaOH in lactated Ringer's solution at 38.4 mL d^−1^) to match the T_3_ solution at the same rate and for the same duration as for T_3_. After 5 days of T_3_ infusion, the duration previously found sufficient to drive maturational changes in cardiomyocyte proliferative phenotype, experiments were concluded at 130 ± 0 dGA.

All ewes were euthanized with an overdose of sodium pentobarbital. Heparin (10,000 U) was administered to the fetal umbilical vein to ensure anticoagulation. A saturated potassium solution was administered to the fetal umbilical vein to arrest the fetal heart in diastole. Fetuses were weighed, their sexes recorded, their hearts dissected in a standardized manner, and their hearts were weighed.

### 2.2 Live cell imaging of fatty acid uptake following in utero T_3_ exposure

Fetal hearts were enzymatically dissociated into individual cells following excision as previously described (Chattergoon et al., 2007; Sundgren et al., 2003). As we have previously established close correspondence between fetal right and left ventricular responses to T_3_ (Chattergoon et al., 2007, 2014; Chattergoon, Louey, et al., 2012), we confined ourselves to use of left ventricular cardiomyocytes or myocardium for all assays. An established protocol in our lab was used with freshly isolated fetal cardiomyocytes to monitor the incorporation of exogenous long‐chain saturated fatty acid into lipid droplets (Drake et al., 2022). We imaged cells with BODIPY™ FL‐C12 (Invitrogen, purchased from Thermo Fisher Scientific, Cat. No. D3822), an 18‐carbon saturated fatty acid comprised of a 12‐carbon chain length saturated fatty acid linked to the fluorophore BODIPY™ (4,4‐difluoro‐3a,4a‐diaza‐s‐indacene). We also imaged with BODIPY™ FL C16 (Invitrogen, purchased from Thermo Fisher Scientific, Cat. No. D3821), a 22‐carbon saturated fatty acid comprised of a 16‐carbon chain length saturated fatty acid linked to BODIPY™. The BODIPY™ fluorophore is an intensely fluorescent, intrinsically lipophilic molecule and permits tracking of exogenous supplied fatty acid. Working solutions of BODIPY™ FL C12 and BODIPY™ FL C16 (both 10 μM) were prepared by diluting a 2.5 mM stock solution dissolved in DMSO into KB solution (1:250; supplemented with 2 mM glutamine, 200 μM sodium pyruvate, 2 mM lactate, 1 mM glucose, 500 μM carnitine) with 0.1% weight per volume fatty acid free bovine serum albumin (BSA; Thermo Fisher, Cat. No. BP9704100) and incubated for 30 min (37°C, in the dark) to allow fatty acid conjugation to BSA.

Freshly isolated left ventricular fetal cardiomyocytes were incubated in supplemented KB in 8‐well μ‐slides (Ibidi USA, Inc., Fitchburg, WI, USA, Cat. No.80821) with 2 μM of BODIPY™ C12 or BODIPY™ C16 for 60 min (39°C, 5% CO_2_) and imaged using the 63× oil lens on a Zeiss 880 LSM with Airyscan software. Z‐stack images were collected after 60 min. For each animal, 20–35 cells were measured in an average of 7 frames (range of 3–12 frames). BODIPY™ was imaged with 488 laser (intensity 0.6, gain 825, digital gain 1.0). A total of 90–130 slices (0.2 μm thick) were acquired per frame (total *z*‐axis thickness of 18–26 μm was greater than any cardiomyocyte diameter).

Z‐stacks were processed (Airyscan) and maximum intensity projections performed in ZEN Black Software (Carl Zeiss Inc., Thornwood, NY, USA). Fiji software was used to analyze all images obtained as described (Drake et al., 2022). Images were enhanced using Enhance Local Contrast (CLAHE: blocksize = 9, histogram = 256, maximum = 4), despeckled, and background subtracted (rolling = 5). Lipid droplet particles were analyzed by filtering for the following parameters: circularity (0.8–1), size (from 0.0314 μm, which is minimal detectable size for the LSM880, to 3 μm, which exceeds the maximum non adipocyte lipid droplet size) (Wang et al., 2013). Images were processed using AutoThreshold (Intermodes dark function) and were converted to a mask for lipid droplets and entire cells. Masks were manually verified to ensure the parameters did not accidentally exclude viable or include non‐viable cells. Lipid droplet number was referenced to total cardiomyocyte area to calculate lipid droplet density.

After data analysis, the output of the green channel was adjusted to improve image quality for visual presentation without changing the ratio of intensities among cardiomyocytes (the input:output linear curve for each image, excluding the scale bar, was shifted from 1:1 to 1:2 without changing the intercept).

### 2.3 Protein expression analysis following in utero T_3_

As biopsies were not frozen from hearts that were dissociated, left ventricular myocardial samples frozen in a previous study from fetuses treated exactly as described above were used for molecular analysis (Chattergoon, Giraud, et al., 2012). Fetuses were randomly allocated to treatment group without knowledge of their sex, and sex was not different between group (vehicle‐infusion three females, five male, 130 ± 0 dGA; T_3_‐infusion five females, three males, 131 ± 0 dGA, *p* = 0.6193).

Frozen left ventricular tissue (25 mg) was homogenized in Upstate RIPA lysis solution (MilliporeSigma, Burlington, VT, USA, Cat. No. 20‐188) with Roche cOmplete™ Mini Protease Inhibitor Cocktail (MilliporeSigma, Cat. No. 11836153001) with a steel bead using the TissueLyser LT (Qiagen, Germantown, MD, USA). Extracted protein was quantified by BCA assay (Pierce, Rockford, IL, USA). Proteins (10 μg per sample) were separated by SDS‐PAGE on a 4%–10% gradient Novex™ Value™ Tris‐glycine gel (Thermo Fisher Scientific) and transferred to a Whatman® Optitran® BA‐S 83 nitrocellulose membrane (Millipore Sigma). Membranes were blocked with 5% milk in 1X Tris‐buffered saline with 0.01% Tween 20 (TBS‐T) buffer for 1 h at room temperature. Membranes were incubated with primary antibodies (1:1000) overnight at 4°C in 1X TBS‐T buffer with 4% BSA.

The primary antibodies used in this study were: anti‐carnitine palmitoyltransferase‐1A rabbit monoclonal, which recognizes endogenous levels of total CPT1A protein and does not cross‐react with CPT1B, CPT1C, or CPT2 (Cell Signaling, Danvers, MA, USA, Cat. No. 12252S Lot No. 3); the anti‐carnitine palmitoyltransferase‐1B rabbit polyclonal antibody recognizes endogenous levels of total CPT1B protein (Cell Signaling, Cat. No. 7074S, Lot No. 29); the anti‐α‐tubulin rabbit monoclonal antibody detects endogenous levels of total α‐tubulin protein and does not cross‐react with recombinant α‐tubulin (Cell Signaling, Cat. No. 7074S, Lot No. 29); the anti‐CD36 rabbit monoclonal antibody (Abcam, Cambridge, MA, USA, Cat. No. ab133625, Lot No. GR281920‐30); the anti‐PPARα rabbit polyclonal (Abcam, Cat. No. ab24509, Lot No. GR333825‐5).

Membranes were washed in large volumes of TBS‐T before exposure to the secondary antibody (1:5000) in TBS‐T with 5% non‐fat milk for 1 h at room temperature. The secondary antibodies goat anti‐rabbit IgG (Cell Signaling, Cat. No. 7074S, Lot No. 29) and horse anti‐mouse IgG were (Cell Signaling, Cat. No. 7076S, Lot No. 32) were horseradish peroxidase‐conjugated. Membranes were then washed in large volumes of TBS‐T three times for 10 min each. Antibody binding was detected using chemiluminescence (SuperSignal, Pierce, IL, USA) and developed on CL‐XPosure TM film (Thermo Fisher Scientific). Protein expression was quantified from a digitized image of the blot using NIH ImageJ (Version 1.53e) (Schneider et al., 2012). Signal density of proteins of interest were normalized to α‐tubulin for each respective sample.

### 2.4 mRNA expression analysis following in utero T_3_

The same set of previously collected biopsies used for western analysis was also used for PCR. Although eight samples were collected per group, only enough tissue from seven animals remained for PCR analysis in the control group. Sex was not different between groups (vehicle‐infusion two females, five male, 130 ± 0 dGA; T_3_‐infusion five females, three males, 131 ± 0 dGA, *p* = 0.3147).

Tissue (25 mg) was homogenized in TRIzol with a steel bead in a TissueLyser LT (Qiagen). RNA was extracted per TRIzol protocol (Invitrogen, Carlsbad, CA, USA). Samples were purified by RNeasy protocol (Qiagen, Cat. No. 74004). cDNA was synthesized using Applied Biosystems™ High‐Capacity cDNA Reverse Transcription Kit with RNase inhibitor (Thermo Fisher Cat. No. 4374966).

Table 1 shows primers for genes used in this study (MX3005P qPCR system, Agilent Technologies Inc.) (Drake et al., 2022). Optimal cycling conditions were experimentally determined for each primer pair. During initial PCR reactions, the size of each PCR product was verified by running it on a 1% agarose gel with a 1Kb + Ladder (Invitrogen). In subsequent runs the melting curve analysis (where temperature is raised from 65 to 95°C at a rate of 0.1°C s^−1^) was used to verify the product. Quantification values were generated from primers with only a single product of expected size that melted at the expected temperature. During the PCR reactions, fluorescence readings were monitored to determine the amount of double‐stranded DNA present. For each unknown sample, relative amounts of transcripts were calculated by standard curve method and referenced to RPL37A.

**TABLE 1:** Nucleic acid sequences for PCR primers.

Gene ID	Forward	Reverse
CD36	CTGGTGGAAATGGTCTTGCT	ATGTGCTGCTGCTTATGGGT
CPT1a	GAGACACCAACCCGAAGATC	GTCTCTGTTCTGCCCTCTCG
CPT1b	GGATGTTTGAGATGCACGGC	GCCAGCGTCTCCATTCGATA
DGAT	AGACACTTCTACAAGCCCATGCTC	AGTGCACTGACCTCATGGAAGA
GPAT	GAAGTGGCTGGTGAGTTAAACCCT	CAGTCTGATCATTGCCGGTGAAAC
HADH	AGAAAACCCCAAGGGTGCTGAT	GCCTCTTGAACAGCTCGTTCTT
IDH	CTGTGTTTGAGACGGCTACAAGGA	CGTAGCTGTGGGATTGGCAATGTT
LCAD	TGAAAGCCGCATTGCCATTGAG	ACTTGGATGGCCCGGTCAATAA
PAP	AGAATGAAGGGAGACTGGGCAAGA	GCAACCAGAGCTCCTTGAATGAGT
PDK4	CCTGTGATGGATAATTCCCG	TTGGTTCCTTGCTTGGGATA
RPL37a	ACCAAGAAGGTCGGAATCGT	GGCACCACCAGCTACTGTTT

The transcripts for the following genes were quantified by PCR: fatty acid transporter CD36, mitochondrial transporters carnitine palmitoyltransferase 1a (CPT1A) and carnitine palmitoyltransferase 1b, (CPT1B), beta oxidation genes long‐chain and very long‐chain acyl‐CoA dehydrogenase (LCAD; VLCAD), hydroxyacyl‐coenzyme A dehydrogenase (HADH), tricarboxylic acid cycle gene isocitrate dehydrogenase (IDH), esterification genes glycerol phosphate acyltransferase (GPAT), phosphatidic acid phosphatase (PAP), and diacylglycerol acyltransferase (DGAT), and reference gene RPL37A.

### 2.5 Cellular respiration in cultured fetal sheep cardiomyocytes

Hearts were collected from nine surgically‐naïve fetuses at 135 ± 0 dGA (five female, four male) and enzymatically dissociated as described above. This developmental age was selected such that fetal T_3_ levels were still low and would not yet have exerted maturational effects on the cardiomyocytes (Jonker & Louey, 2016). Cardiomyocytes were preplated and cultured in 10% serum media as previously described (Chattergoon et al., 2007; Sundgren et al., 2003), but in T‐75 flasks (7–12 million/flask) until ~70% confluent (typically 2–3 days) instead of in 35 mm plates. Media was replaced with fresh serum media 24 h before cells were released from the flask with trypsin (0.25% trypsin–EDTA) and resuspended in cryoprotectant (10% DMSO, 90% FBS) at 1–10 million viable cells per ml. Vials were gradually cooled at −1°C min^−1^ in a Mr. Frosty Freezing Container (Thermo Fisher, Cat. No. 5100‐0001) in a − 80°C freezer for up to 48 h, and then transferred to liquid nitrogen storage. To conduct experiments, cryotubes containing cardiomyocytes were retrieved from liquid nitrogen storage, warmed for 30 s in a 39°C water bath, and thawed contents added to sterile warmed serum‐supplemented media (Chattergoon et al., 2014; O'Tierney et al., 2010). Cell viability and number were determined by Trypan blue exclusion (MilliporeSigma, Cat. No. T8154). Passage 1 cardiomyocytes were seeded at 20,000 cells per well in serum‐supplemented media for 24 h before being changed to serum free (SF) media. After 24 h cardiomyocytes were subjected to experimental conditions. All cells were treated with T_3_ or vehicle (1.25 μM NaOH) for 24 h in serum‐free media. Some cells were also treated for 24 h with PA (100 or 200 μM, Thermo Fisher, Cat. No. 129702500) with BSA (6:1 ratio) and 50 μM carnitine supplementation, or vehicle without PA.

T_3_ doses were given to approximate physiological concentrations at various stages of development. The lowest dose of T_3_ (0.75 nM) in the metabolic assay is about equivalent to the total circulating T_3_ in the ovine fetus 5 days before birth, just before the dramatic rise in T_3_ levels begins (Jonker & Louey, 2016). The middle dose of T_3_ (1.5 nM) is approximately equal to the level of total T_3_ in the ovine fetus 2 days before birth, which is also the level of total T_3_ achieved with the in vivo infusion (Chattergoon, Giraud, et al., 2012). The highest dose of T_3_ in the metabolic study (10 nM) is only somewhat higher than the peak of total T_3_ levels in the newborn lamb.

One hour before the assay, the cells were washed twice and left to equilibrate for 1 h in non‐buffered base medium (Agilent Technologies Inc.) in room air at 39°C. The base medium was supplemented with 2 mM glutamine, 200 μM sodium pyruvate, 2% FBS, 1 mM glucose, and 2 mM l‐lactate. For those cells that were to receive PA (100 μM) acutely as a metabolic substrate, media was replaced with supplemented base media with PA 30 min prior to the assay.

Basal and maximal oxygen consumption rates (OCR) were measured in a “mitochondrial stress test” with the XFe96 Seahorse Extracellular Flux Analyzer (Figure S1; Agilent Technologies Inc., Santa Clara, CA, USA). Following the calibration and equilibration period, 3 min measurements were taken three times alternating with 3 min of mixing at (1) basal conditions (reflective of basal respiration); (2) after introduction of the ATP synthase inhibitor oligomycin, 1.5 μM (reflective of proton leak), (3) after introduction of the mitochondrial uncoupling agent carbonyl cyanide‐p‐trifluoromethoxyphenylhydrazone (FCCP; 2.7 μM; reflective of maximal respiration); and (4) after introduction of the complex I and cytochrome c reductase inhibitors rotenone‐antimycin A, 1 μM (reflective of non‐mitochondrial respiration). Treatments and measurements were simultaneous in each well. The concentrations of the compounds (and cardiomyocyte number) were determined by titrating for their maximal bioenergetic effect.

Cells from 6 to 9 fetuses were used for assays. There was a minimum of five replicates per treatment condition. The assay was replicated two times to test reproducibility. Data from the mitochondrial stress test conditions were normalized to the basal respiration level to compare maximal OCR (as a percentage of basal respiration) between treatment groups. There are competing considerations when choosing how to analyze this data. We validated our findings by analysis of OCR following quantification of protein concentration by BCA assay in every well for all subjects except one for which the plate was disposed of accidentally.

### 2.6 Statistical analysis

Data were analyzed and graphed in GraphPad Prism version 9.5.0 (GraphPad Software, San Diego, California USA, www.graphpad.com). Physiological parameters measured at baseline (Day 0) and on the final experimental day (Day 5) were analyzed by repeated‐measures (by time) 2‐way analysis of variance (ANOVA). Fetal sex distribution was analyzed by Fisher's exact test. Cellular respiration data were analyzed by repeated‐measures (by fetus as source of cells) one‐way ANOVA (or mixed‐effects model) followed, if warranted, by Tukey's multiple comparisons test. All other data were analyzed with Student's unpaired, two‐tailed *t*‐test. Normality of data distribution was assessed by the D'Agostino‐Pearson test, and outliers were identified by the ROUT method (Motulsky & Brown, 2006). Statistical significance was determined at *p* < 0.05. All values of *p* < 0.10 are reported. Data are displayed as mean ± standard deviation (SD).

## 3 RESULTS

### 3.1 In utero effects of T_3_ administration

Vascular pressures and heart rate were not different between groups following 5 days of T_3_ (54 μg d^−1^) or vehicle treatment (Table 2). Arterial pressure increased 7% over the study period similarly between treatment groups (*p* < 0.0001, Table 2). Arterial pH, partial pressures of carbon dioxide (pco_2_) and oxygen (po_2_) were not different between groups or with advancing age. Body weight at necropsy was similar between groups (Table 3). Heart weight was 17% greater in the fetuses infused with T_3_ versus those infused with vehicle (*p* = 0.0398), however the heart to body weight ratio was not different between groups.

**TABLE 2:** Hemodynamic and arterial blood gas parameters.

	Vehicle‐infused		T_3_‐infused	
	Baseline	Final	Baseline	Final
Arterial pressure (mmHg)^a^	40.4 ± 2.0	42.4 ± 2.9	40.5 ± 1.2	44.2 ± 1.6
Venous pressure (mmHg)	2.7 ± 1.0	2.5 ± 1.3	2.0 ± 0.8	2.5 ± 0.8
Heart rate (bpm)	171 ± 15	164 ± 15	165 ± 15	165 ± 15
pH	7.36 ± 0.02	7.35 ± 0.02	7.36 ± 0.01	7.36 ± 0.02
pco_2_ (mmHg)	50.2 ± 2.3	47.9 ± 1.9	49.6 ± 2.0	49.5 ± 2.1
po_2_ (mmHg)	22.6 ± 2.2	23.1 ± 1.6	23.2 ± 2.9	21.9 ± 2.5

*Note*: *n* = 7 per group. Data shown as mean ± SD. Analyzed by 2‐way ANOVA.

^a^ *p* < 0.0001 different by main effect of time in the absence of interaction between treatment and time.

**TABLE 3:** Body and heart weights.

	Control	T_3_‐infused
Body (kg)	4.3 ± 0.6	4.8 ± 0.6
Heart (g)	26.8 ± 4.9	31.3 ± 1.8^a^
Heart body^−1^ (g kg^−1^)	6.2 ± 0.6	6.6 ± 0.6

*Note*: *n* = 7 per group. Data shown as mean ± SD. Analyzed by Student's unpaired *T*‐test.

^a^ *p* = 0.0398 versus Control.

### 3.2 The effect of in utero T_3_ on lipid uptake in ex vivo cardiomyocytes

**FIGURE 1:**
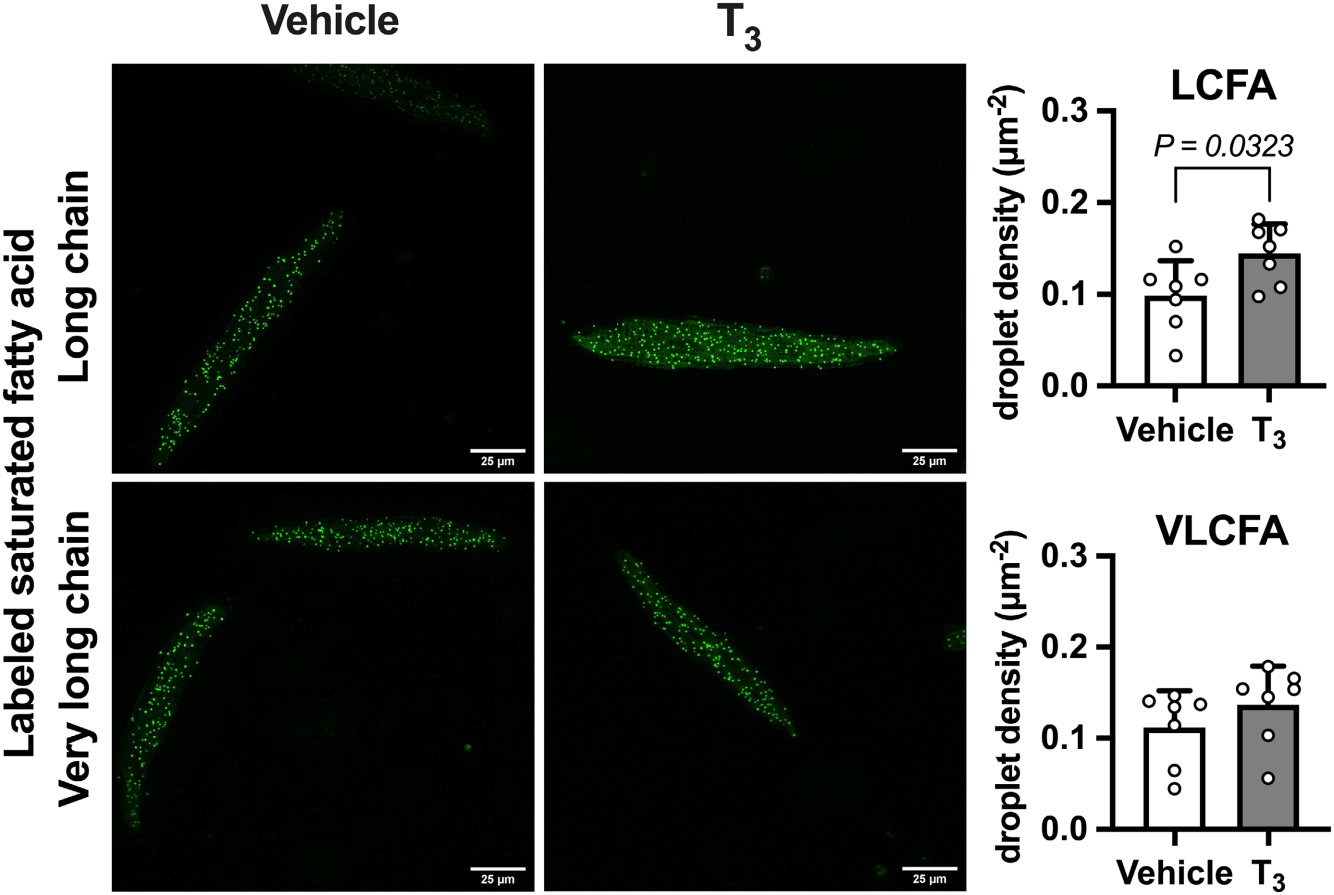
Lipid uptake and droplet formation in isolated cardiomyocytes. On the left are representative images of BODIPY™‐labeled fatty acid incorporation into cellular lipid droplets (green; magnification 630×). Summarized data are shown on the right. There are a greater number of labeled long‐chain fatty acid (LCFA) lipid droplets per cell area (upper panels) in left ventricular cardiomyocytes from T_3_‐infused fetuses compared to vehicle‐infused fetuses. There are no differences in lipid droplet density for very long‐chain (VLCFA; lower panels). Scale bars = 25 μm. *n* = 7 fetuses per group. Mean ± SD. Treatments compared by Student's unpaired *t*‐test.

Following 1 h of incubation with BODIPY™‐labeled long‐chain fatty acid (LCFA), there were 42% more droplets per cell area in cardiomyocytes from T_3_‐infused fetuses than from vehicle‐infused fetuses (*p* < 0.0323; Figure 1). When incubated with very long‐chain fatty acid (VLCFA) instead, there was no difference in lipid droplet density.

### 3.3 Myocardial metabolic gene mRNA and protein expression levels following in utero T_3_

**FIGURE 2:**
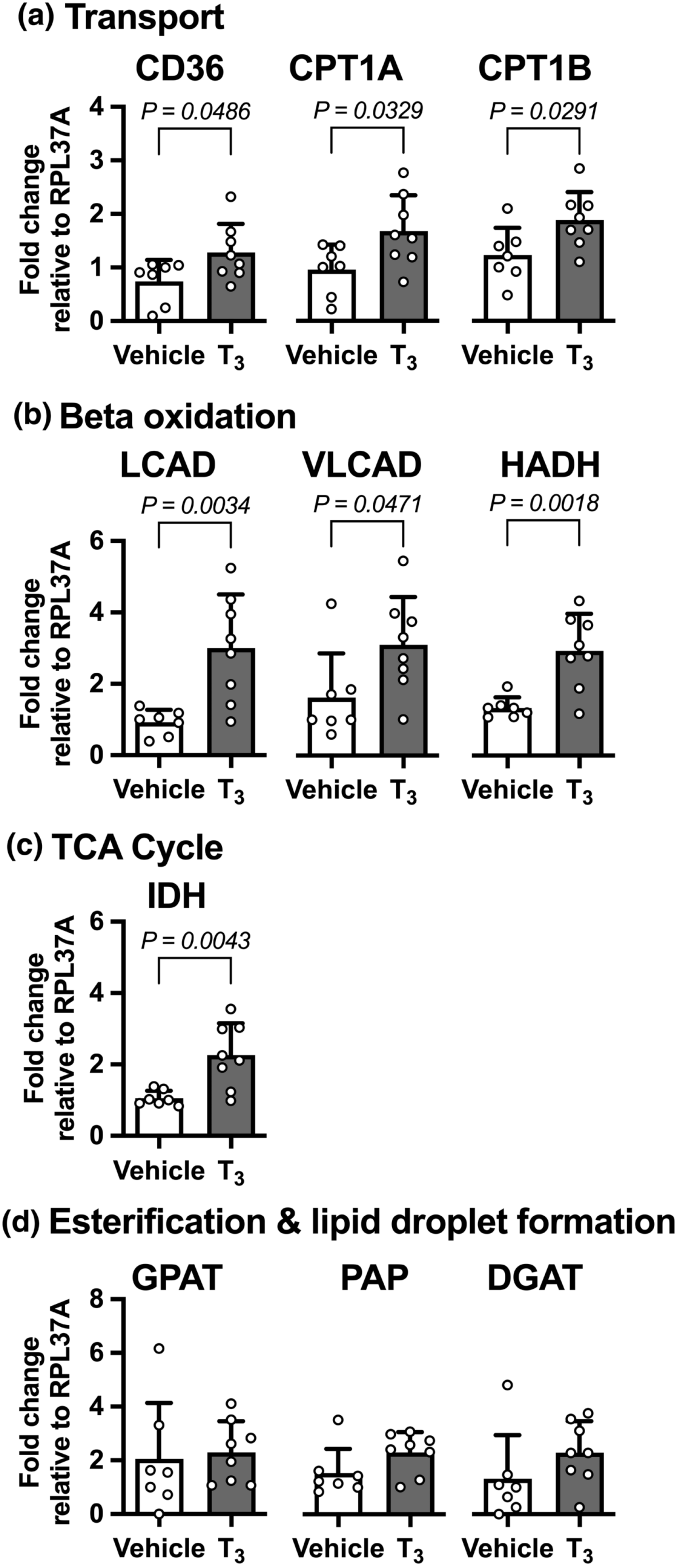
Left ventricular metabolic gene mRNA expression levels following 5 days of exposure to elevated T_3_ in utero. Expression levels of mRNA were measured in left ventricular myocardium from fetuses that had received an intravenous infusion of T_3_ (54 μg d^−1^; *n* = 8) or vehicle (*n* = 7) for 5 days. Following T_3_ infusion there were increases in mRNA levels for genes involved in (a) fatty acid transporter, (b) beta oxidation, and (c) the citric acid cycle, but not (d) fatty acid esterification and lipid droplet formation. Mean ± SD. Treatments compared by Student's unpaired *t*‐test.

Elements of the system for cellular use of fatty acids were upregulated in hearts of fetuses that received in vivo infusion of T_3_ compared to those that received vehicle (Figure 2). mRNA levels were upregulated for key molecules responsible for transport of fatty acids across lipid membranes (Figure 2a): CD36 (72%; *p* = 0.0486), and the mitochondrial transporters CPT1A (75%; *p* = 0.0329), and CPT1B (53%; *p* = 0.0291).

In utero exposure to T_3_ also increased mRNA expression levels of mitochondrial genes related to beta oxidation (Figure 2b). LCAD, which is responsible for the first step of breaking down long‐chain fatty acids, was increased by 226% (*p* = 0.0034). VLCAD, which is responsible for the first step of breaking down very long‐chain fatty acids, was increased by 91% (*p* = 0.0471). HADH, which metabolizes medium‐ and short‐chain fatty acids, was increased by 119% (*p* = 0.0018).

IDH, responsible for a key step in the tricarboxylic acid cycle, was increased 114% by fetal T_3_ infusion (*p* = 0.0043; Figure 2c). Fetal infusion of T_3_ had no effect on the mRNA expression levels of genes involved in esterification and lipid droplet formation, GPAT, PAP (*p* = 0.0991), and DGAT (Figure 2d).

In contrast to increased mRNA expression levels, protein levels were unchanged by fetal in vivo T_3_ infusion versus control for CD36 (0.52 ± 0.15 vs. 0.90 ± 0.23), CPT1A (0.47 ± 0.16 vs. 0.58 ± 0.30), CPT1B (0.40 ± 0.09 vs. 0.67 ± 0.18) or PPARα (0.50 ± 0.23 vs. 1.17 ± 0.61; Figure S2).

### 3.4 Myocardial regulatory gene mRNA expression levels following in utero T_3_

**FIGURE 3:**
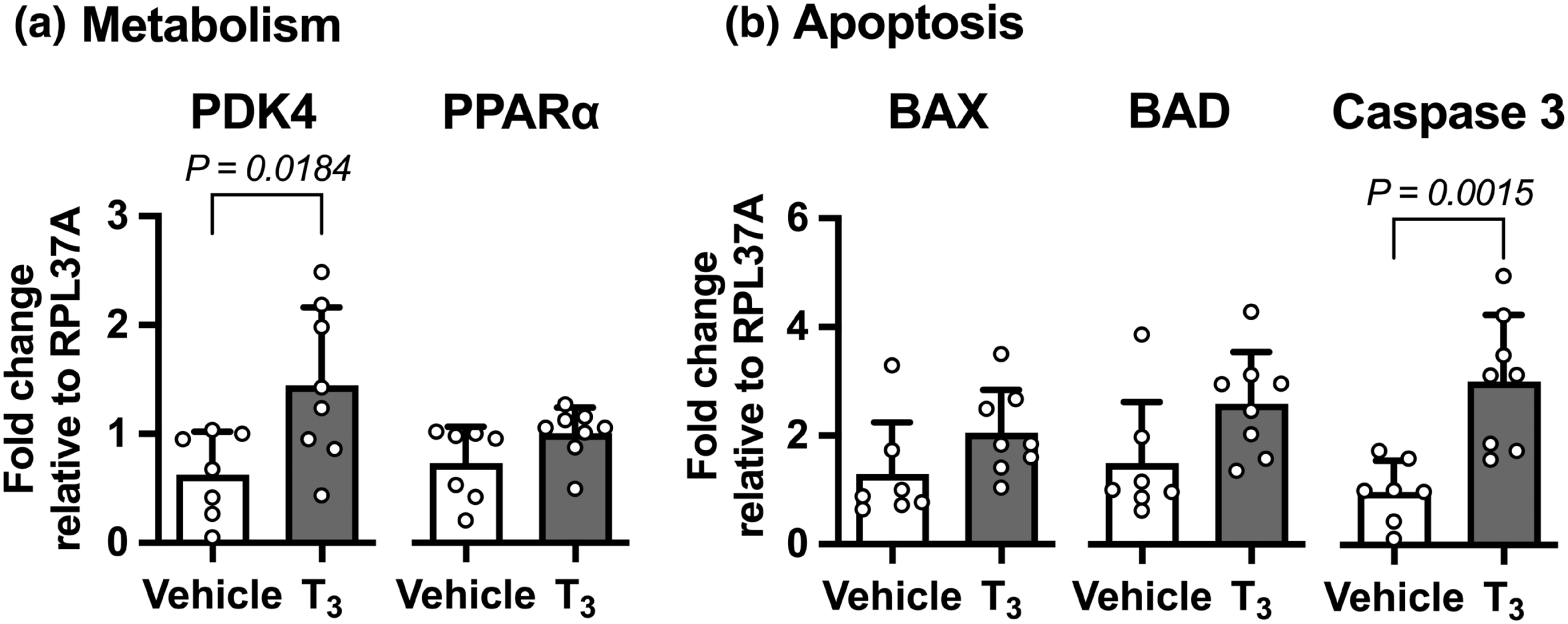
Left ventricular regulatory gene mRNA expression levels following 5 days of exposure to elevated T_3_ in utero. Expression levels of mRNA were measured in left ventricular myocardium from fetuses that had received an intravenous infusion of T_3_ (54 μg d^−1^; *n* = 8) or vehicle (*n* = 7) for 5 days. Following T_3_ infusion (a) the metabolic regulator PDK for was elevated by T_3_ exposure, but the transcription factor PPARα was not. (b) Apoptosis regulators were not affected, but caspase 3 was elevated. Mean ± SD. Treatments compared by Student's unpaired *t*‐test.

mRNA of metabolic regulatory molecules was also assessed (Figure 3a). In vivo exposure to T_3_ increased expression of PDK4 by 130% (*p* = 0.0184), which can upregulate fatty acid utilization by inhibiting pyruvate dehydrogenase (Zhang et al., 2014). However, the T_3_‐stimulated 38% increase in PPARα, which can upregulate fatty acid catabolism through transcription factor activity, was not significant (*p* = 0.0863).

We measured mRNA expression levels pro‐apoptotic genes following in utero T_3_ exposure (Figure 3b). We found apoptosis promotor BAX unchanged, and the 74% increase in BAD was not significant (*p* = 0.0611). However, mRNA for the apoptosis effector caspase 3 was increased 207% (*p* = 0.0015).

### 3.5 The effects of T_3_ and PA on maximal respiration in cultured cardiomyocytes

**FIGURE 4:**
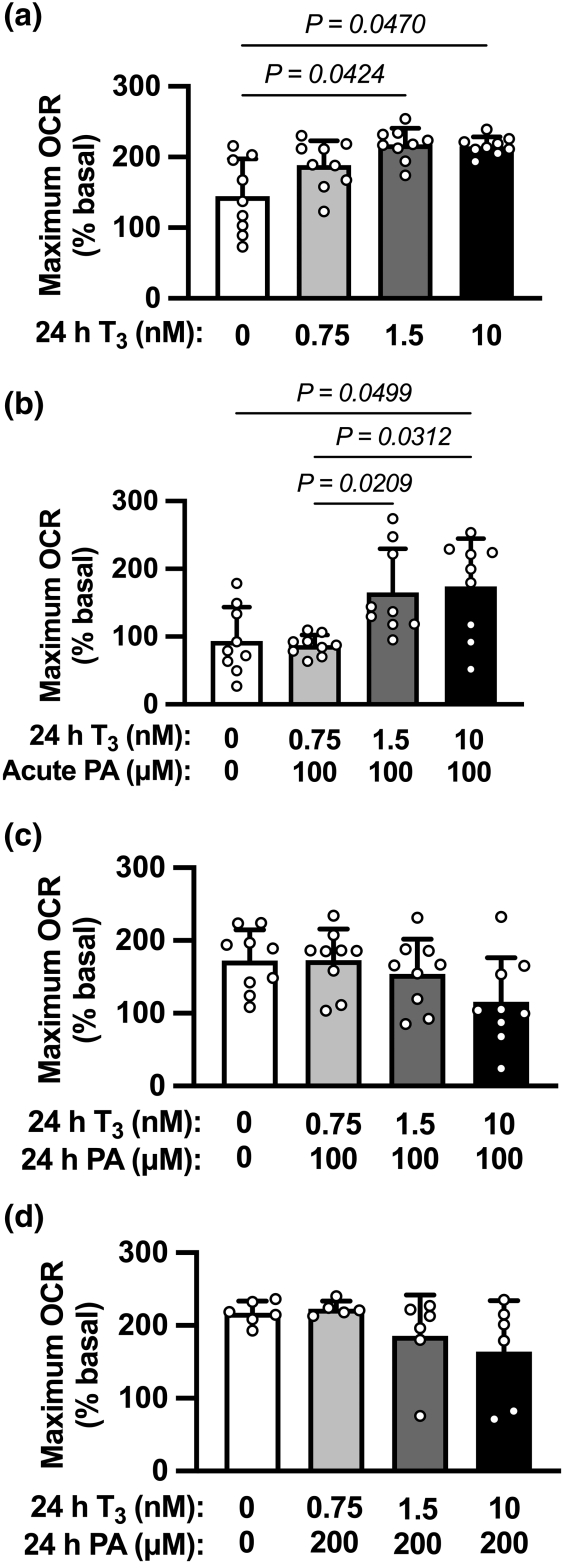
The effects of T_3_ and PA on oxygen consumption rates in fetal cardiomyocytes. Oxygen consumption rates (OCR) were measured in cultured fetal cardiomyocytes using the Seahorse Extracellular Flux Analyzer. (a) Treatment with T_3_ (24 h; all doses) increased maximal OCR. A threshold rather than a dose‐dependent effect was observed. (b) Adding palmitic acid (PA) as an energetic substrate at the time of analysis yielded a similar result, but shifted the T_3_ threshold (0.75 nM to 1.5 nM). In contrast, 24 h treatment of cells with (c) 100 μM PA or (d) 200 μM PA eliminated the T_3_‐stimulated increase in maximal OCR, suggesting suppression of oxygen consumption by PA. *n* = 9 fetuses per group (Panels a–c), *n* = 6 (Panel D, except *n* = 5 for T_3_ 0.75 nM). Groups were compared by 1‐way ANOVA followed, if warranted, by Tukey's multiple comparisons test. Mean ± SD.

Control cardiomyocytes were compared with those exposed T_3_ for 24 h (Figure 4a; ANOVA *p* = 0.0039). Maximal OCR in cells treated with either 1.5 or 10 nM T_3_ was ~50% higher compared to cells that received vehicle (*p* = 0.0424 and *p* = 0.0470, respectively). The respiratory response to T_3_ tended to be different between 0.75 mM and 1.5 nM T_3_ (*p* = 0.0835).

T_3_ continued to increase maximal OCR when PA (100 μM) was provided acutely as a metabolic substrate (Figure 4b; ANOVA *p* = 0.0004 There was a threshold effect in the respiratory response to T_3_ in the presence of acute PA (0.75 mM vs. 1.5 nM T_3_ *p* = 0.0209, and 0.75 mM vs. 10 nM T_3_ *p* = 0.0312).

In contrast, exposure to PA (100 or 200 μM) for 24 h prior to metabolic measurements tended to eliminate the stimulatory effect of T_3_ on maximal OCR (Figure 4c,d; ANOVA *p* = 0.0726 and *p* = 0.0887, respectively).

**FIGURE 5:**
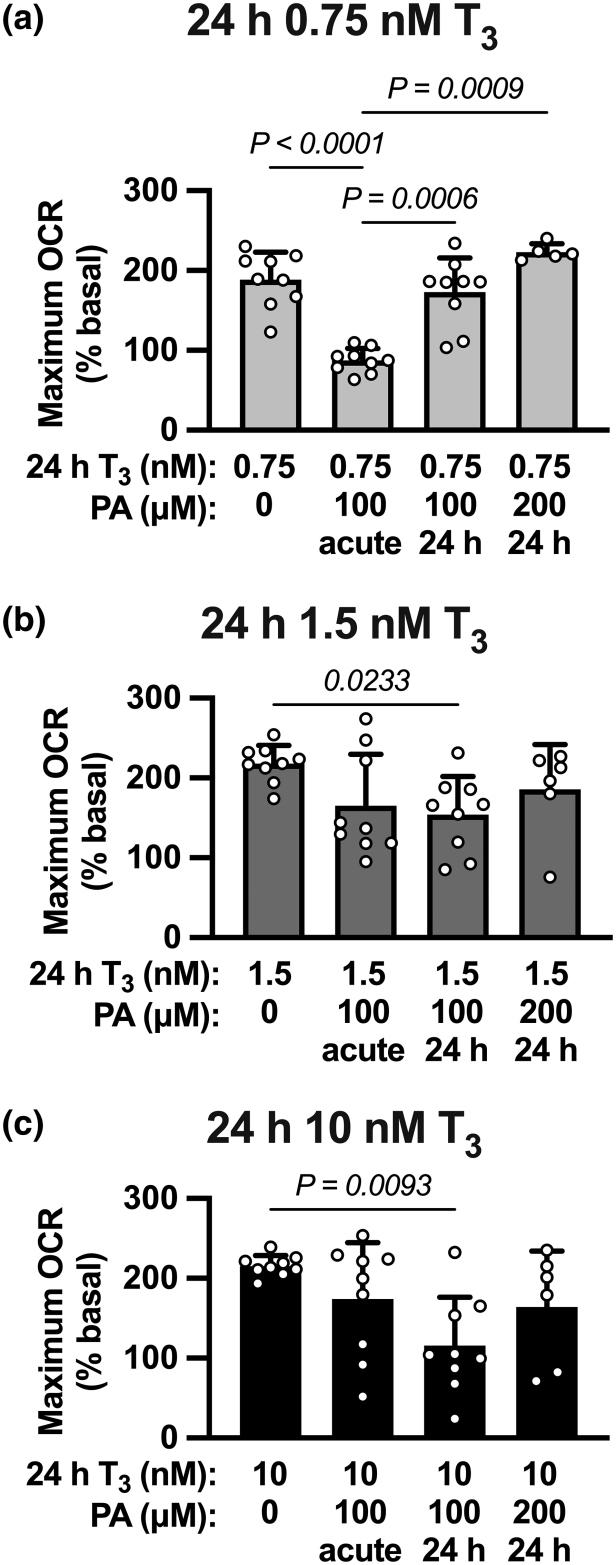
The role of palmitic acid on maximal oxygen consumption rate in the presence of T_3_. Maximal oxygen consumption rate (OCR) was compared in the presence of varying doses of T_3_ across all exposures to palmitic acid (PA). (a) Acute PA (100 μM) suppressed maximum OCR compared to control cells that only received 0.75 nM T_3_. However, maximal OCR following 24 h exposure to PA (100 or 200 μM) was similar to that in control cells, suggesting cellular adaptation to lipid as a substrate at this low level of T_3_. At (b) 1.5 nM T_3_, and (c) 10 nM T_3_ there were PA dose‐dependent depressions in maximal OCR with chronic PA exposure. *n* = 9 fetuses per group, except *n* = 6 for the groups treated with 200 μM PA and *n* = 5 for 200 μM PA + T_3_ 0.75 nM. Groups were compared by 1‐way ANOVA followed by Tukey's multiple comparisons test. Mean ± SD.

To better understand the role of PA exposure on fetal cardiomyocyte respiration, maximal OCR was compared in cells that were exposed to similar doses of T_3_. At 0.75 nM T_3_ (Figure 5a; ANOVA *p* < 0.0001), acute exposure to PA (100 μM) depressed maximal OCR ~54% compared to no PA (P < 0.0001), as well as compared to those exposed to 24 h of PA (vs. 100 μM, *p* = 0.0006; vs. 200 μM, *p* = 0.0009). At higher levels of T_3_, chronic exposure to PA decreased maximal OCR (Figure 5b,c; 1.5 nM T_3_ ANOVA *p* = 0.0349; 10 nM T_3_ ANOVA *p* = 0.0226). 24 h of 100 μM PA depressed respiration compared to no PA at both 1.5 nM T_3_ (*p* = 0.0233) and 10 nM T_3_ (*p* = 0.0093).

Proton leak, normalized to basal respiration, was not affected by T_3_ treatment or PA availability in fetal cardiomyocytes (data not shown).

Protein concentrations in assay wells were not different by T_3_ level, or by or PA treatment (*p* = 0.0595; Figure S3). Data trends for maximal OCR were similar when normalized to protein concentration instead of basal respiration (Figure S4).

## 4 DISCUSSION

This study provides evidence for three conclusions: (1) T_3_ exposure in utero leads to cardiomyocytes that increase lipid uptake and droplet formation when exposed to long‐chain fatty acid, (2) raising circulating T_3_ levels in utero also stimulates mRNA expression of lipid‐handling systems of the fetal myocardium as expected during maturation, (3) T_3_ treatment in vitro raises maximal oxygen consumption rates, but PA suppresses maximal oxygen consumption at any level of T_3_. These conclusions validate our hypothesis that T_3_ augments free fatty acid uptake in fetal cardiomyocytes, and that it increases maximal oxidative capacity. However, the regulatory role of PA on fetal cardiomyocyte metabolism was more complex than the stimulatory action hypothesized.

### 4.1 Role of thyroid hormone

It is well known that among developing mammals the myocardium switches from a carbohydrate‐dominated process for generating ATP to lipid‐dominated metabolism using beta oxidation to generate ATP during the perinatal period (Bartelds et al., 2000; Fisher et al., 1980; Lopaschuk & Jaswal, 2010). The biochemical machinery required for the switch is assembled over the course of weeks before birth, and the fetal myocardium is capable of at least partially using fatty acids (Bartelds et al., 2000). In the days prior to birth, fetal T_3_ levels rise to the same level achieved by the fetal infusion protocol used in this study (Chattergoon, Giraud, et al., 2012; Jonker & Louey, 2016). In agreement with previous investigations (Graham & Huang, 2021; Sayre & Lechleiter, 2012), this study suggests that rising T_3_ levels in utero contribute to increased maximal capacity for oxidative metabolism, concurrent with the other maturational changes stimulated by T_3_. Cardiac metabolic dysfunction may be a consequence in preterm babies that do not experience a normal rise in T_3_ (Eerdekens et al., 2019), as well as fetuses affected by thyroid disorders during pregnancy (Mallawa Kankanamalage et al., 2021; Maulik et al., 2022). The normal function of the fetal heart is also disrupted by experimental thyroidectomy, including impairing the normal age‐related decline in cardiac glycogen storage (Forhead et al., 2009).

We assessed the expression levels of key enzymes and regulators of lipid utilization in myocardium of fetuses exposed to early increase in T_3_ and found that mRNA levels of these systems were broadly elevated, largely similar to changes in expression during perinatal cardiac maturation (Drake et al., 2023). It is interesting that these changes were not translated into increased protein expression of fatty acid transporters. The accelerated fatty acid incorporation into lipid droplets in isolated cardiomyocytes, and elevated maximal oxygen consumption, suggest that protein functions were altered. It may be that changes in protein expression would become evident after longer exposure, or that the effect of T_3_ was via post‐translational regulation.

Lipid droplets are important organelles that provide storage for neutral lipids that may be metabolized when needed at a later time and may protect against lipotoxicity (Goldberg et al., 2018; Krahmer et al., 2013; Listenberger et al., 2003). However, the interpretation of changes in size and number of droplets is often difficult. Large numbers of enlarged lipid droplets are associated with many organ‐based pathologies including cardiac steatosis and cardiomyopathy. Lipid droplet dysfunction is associated with metabolic stress as found in heart failure (O'Donnell et al., 2008). In this study, long‐chain lipid droplet density was greater in cardiomyocytes treated with T_3_ in utero. In contrast, though, neonatal cardiomyocytes demonstrated the same density but larger droplets compared to fetal cardiomyocytes (Drake et al., n.d.). We speculate that the increase in the numbers of lipid droplets formed from the uptake of long‐chain fatty acids reflects an augmentation of the lipid uptake and storage systems within the normal myocyte although it does not exactly reflect the normal developmental change. Because very long‐chain fatty acids (≥22 chains) will, because of their size, enter the cell more slowly than the 18‐carbon chain fatty acids (Kolahi et al., 2018), it is not surprising that the uptake and storage of the larger species was increased on the average but did not reach significance under the time constraints and numbers of samples included in this study.

### 4.2 Role of palmitic acid

We hypothesized that PA would augment the maturational process begun by T_3_, reasoning that the presence of a fatty acid would itself signal that beta oxidation had become an important necessity to the heart. A hypothesis reflecting the dark side of PA would have also been legitimate, as PA is known to promote apoptosis among neonatal and adult cardiomyocytes (Oh et al., 2014, 2019; Wang et al., 2017; Ying et al., 2015). Thus, one might speculate in fetal cardiomyocytes that toxicity associated with PA exposure would exert a suppressive influence on oxygen consumption rate and eventual promotion of cell death via apoptosis. We found some evidence that this is true: while acute exposure to PA did not impair the augmentation of maximal oxidation by increasing levels of T_3_ (Figure 4b), it did impair maximal oxidation at a fixed dose of T_3_ (Figure 5a). Further, longer‐term exposure to PA abrogated the elevation in oxygen consumption rate stimulated by 1.5 and 10 nM T_3_ (Figure 5b,c). Others have shown that PA causes pro‐inflammatory changes in cardiomyocytes as the concentration increases above optimal levels (Joseph et al., 2016, 2017). One detrimental effect of palmitate is a decrease the electrical potential of the inner mitochondrial membrane, suppression of oxidative phosphorylation, and generation of reactive oxygen species.

### 4.3 Limitations of the study

A limitation of this study is that we focused exclusively on the near‐term fetus, at 85%–90% of gestation. Studies at midgestation may provide important insight into the metabolic capacities of less mature premature infants, who experience untimely exposure to T_3_ (following antenatal corticosteroid therapy) and high‐lipid nutrition (Franko et al., 2007; Ikegami et al., 1997). Another limitation of our study was the inability to study protein levels of all of the genes for which we measured mRNA levels. This was in part due to limited availability of reliable, specific antibodies, but even for those proteins that we did study, we did not find an effect of T_3_ exposure. The disconnect between our mRNA, protein, and functional data represents a level of regulation that we have not identified.

### 4.4 Summary and conclusions

In fetal sheep, T_3_ contributes to metabolic maturation of cardiomyocytes in preparation for use of fatty acids as a fuel source at birth. Following exposure to physiologically‐relevant levels of T_3_, fetal cardiomyocytes upregulate mRNA expression of a broad panel of genes important to fatty acid utilization, and have a greater capacity for long‐chain fatty acid uptake and lipid droplet formation. However, rather than contributing to metabolic maturation as hypothesized, exposure of immature cardiomyocytes to PA may impair cellular oxidative capacity.

## AUTHOR CONTRIBUTIONS

Chattergoon, Jonker, and Thornburg conceived the study, analyzed the data, and drafted the manuscript. Chattergoon, Jonker, and Louey carried out the experiments. All authors critically revised the manuscript and approved the final version.

## CONFLICT OF INTEREST STATEMENT

None of the authors have any competing interest to declare.

## Supporting information


Figure S1.

Figure S2.

Figure S3.

Figure S4.

## Data Availability

Data are available upon request of the authors.
