# Supplementary material for: Thyroid hormone increases fatty acid use in fetal ovine cardiac myocytes
Source: Physiol Rep. 2023 Nov 27;11(22):e15865. doi: 10.14814/phy2.15865 (PMC10680578; doi:10.14814/phy2.15865)
Supplement: Supplementary file 1 — Figure S1. [file PHY2-11-e15865-s004.pdf]

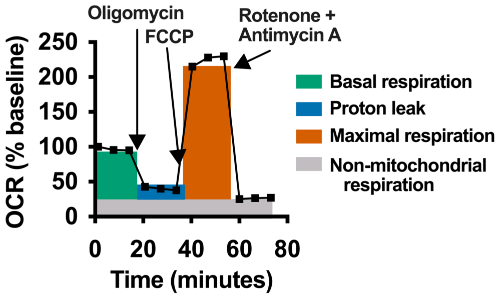

Figure S1. A schematic of an XFe96 Seahorse Extracellular Flux Analyzer mitochondrial stress test. Mitochondrial respiration is measured in cardiomyocytes by measuring oxygen consumption rate (OCR). Basal respiration, proton leak, maximal respiration, and non-mitochondrial respiration can be measured by adding, in succession, oligomycin (an ATP synthase inhibitor), carbonyl cyanide-p-trifluoromethoxyphenylhydrazone (FCCP; a proton ionophore which uncouples oxygen utilization from ATP production), and antimycin A with rotenone (electron transport chain inhibitors). Three measurements are taken at each condition.
