# Supplementary material for: Thyroid hormone increases fatty acid use in fetal ovine cardiac myocytes
Source: Physiol Rep. 2023 Nov 27;11(22):e15865. doi: 10.14814/phy2.15865 (PMC10680578; doi:10.14814/phy2.15865)
Supplement: Supplementary file 2 — Figure S2. [file PHY2-11-e15865-s003.pdf]

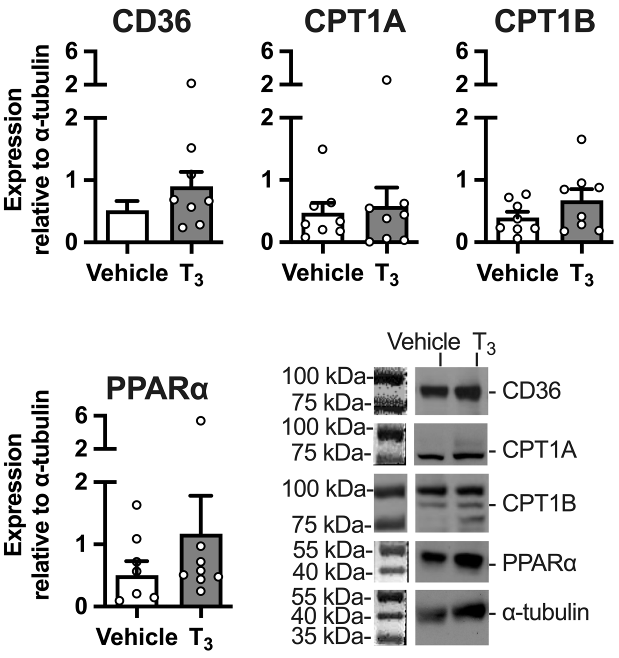

Figure S2. Left ventricular metabolic protein expression levels following 5 days of exposure to elevated T_3 *in utero.* Expression levels of protein were measured in left ventricular myocardium from fetuses that had received an intravenous infusion of T_3 (54 µg d^{-1}; n=8) or vehicle (n=8) for 5 days. Results were analyzed by Student's unpaired t-test, no differences were detected. Mean ± SD.
