# Supplementary material for: Thyroid hormone increases fatty acid use in fetal ovine cardiac myocytes
Source: Physiol Rep. 2023 Nov 27;11(22):e15865. doi: 10.14814/phy2.15865 (PMC10680578; doi:10.14814/phy2.15865)
Supplement: Supplementary file 3 — Figure S3. [file PHY2-11-e15865-s002.pdf]

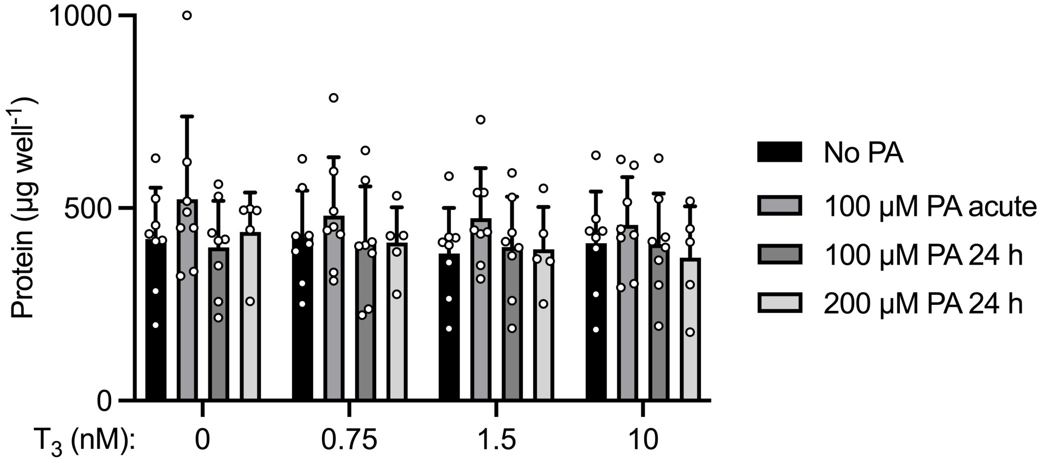

Figure S3. Protein concentration in Seahorse metabolic analyzer wells. Protein concentrations were measured (BCA assay) as an estimate of cellular contents in each treatment well of the Seahorse metabolic analyzer assay plates. Results were measured by 2-way ANOVA, no differences were detected (main effect of palmitic acid (PA) treatment $P=0.0595$). For all groups $n=8$, except 200 µM PA $n=5$. Mean ± SD.
