# Supplementary material for: Thyroid hormone increases fatty acid use in fetal ovine cardiac myocytes
Source: Physiol Rep. 2023 Nov 27;11(22):e15865. doi: 10.14814/phy2.15865 (PMC10680578; doi:10.14814/phy2.15865)
Supplement: Supplementary file 4 — Figure S4. [file PHY2-11-e15865-s001.pdf]

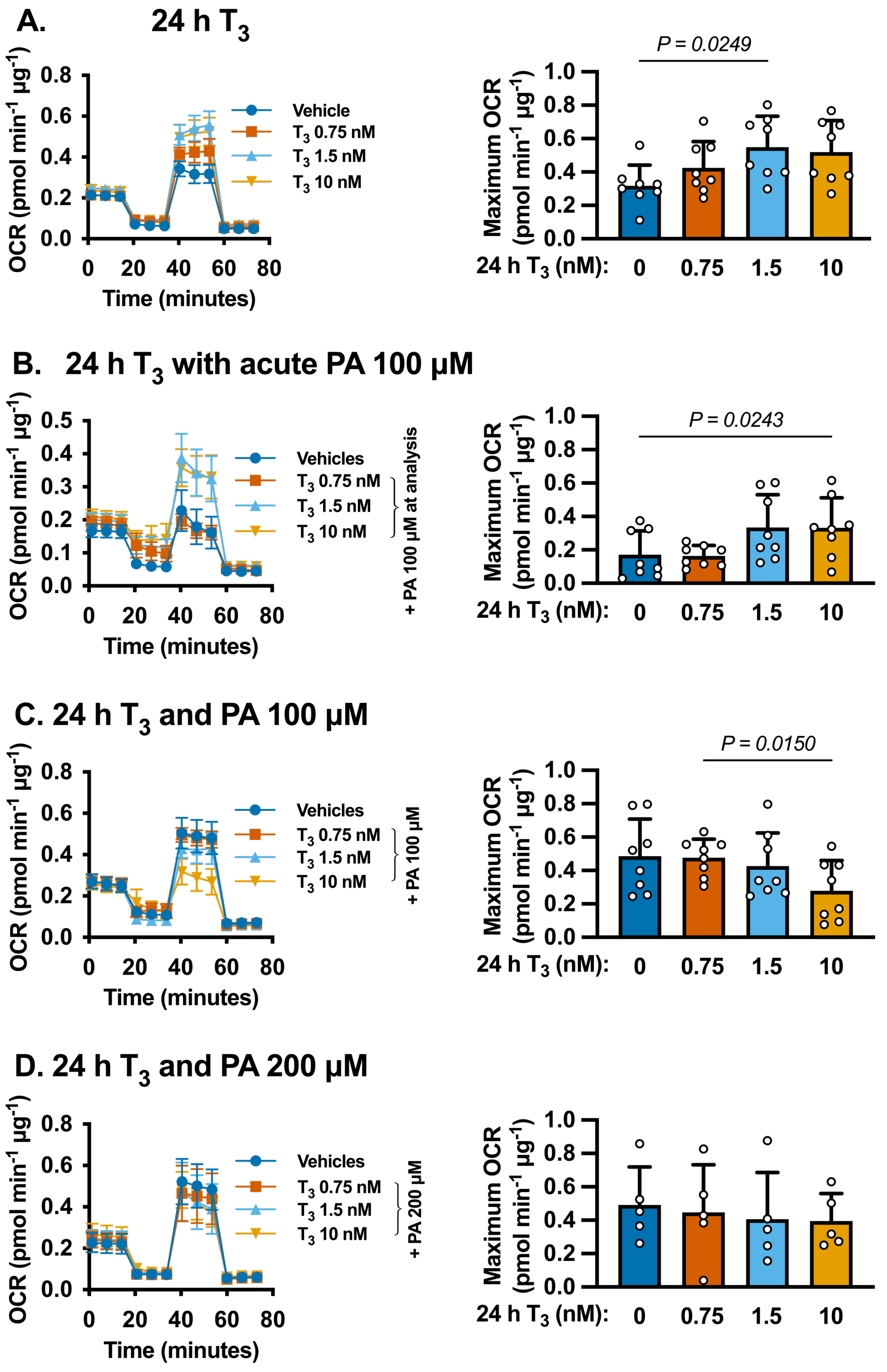


**Figure S4. Seahorse stress tests showing oxygen consumption rate (OCR) normalized to protein concentration.** Oxygen consumption rates (OCR) were measured in cultured fetal cardiomyocytes using the Seahorse Extracellular Flux Analyzer. Condition plots were normalized to protein concentration within the assay well. A) Treatment with $T_3$ or vehicle (24 h). B) Treatment with $T_3$ for 24 h, with palmitic acid (PA; 100 µM) added as an energetic substrate at the time of analysis. D) Treatment with both $T_3$ and PA (100 µM) for 24 h. D) Treatment with both $T_3$ and PA (200 µM) for 24 h. n=8 fetuses per group (Panels A-C), n=5 (Panel D). Groups were compared by 1-way ANOVA followed, if warranted, by Tukey's multiple comparisons test. Mean ± SD.
